# Persistent Orofacial Pain Attendances at General Medical Practitioners

**DOI:** 10.1177/00220345221128226

**Published:** 2022-10-30

**Authors:** C.C. Currie, J. Palmer, S.J. Stone, P. Brocklehurst, V.R. Aggarwal, P.J. Dorman, M.S. Pearce, J. Durham

**Affiliations:** 1 School of Dental Sciences, Newcastle University, Newcastle upon Tyne, UK; 2 Newcastle Upon Tyne NHS Foundation Trust, Newcastle upon Tyne, UK; 3 Public Health Wales, Bangor, Wales; 4 School of Dentistry, University of Leeds, Leeds, UK; 5 Population Health Sciences Institute, Newcastle University, Newcastle upon Tyne, UK

**Keywords:** 6 MeSH: temporomandibular joint disorders, facial pain, health services research, burning mouth syndrome, temporomandibular joint dysfunction syndrome, neuralgia

## Abstract

Patients with persistent orofacial pain (POFP) can go through complex care pathways to receive a diagnosis and management, which can negatively affect their pain. This study aimed to describe 44-y trends in attendances at Welsh medical practices for POFP and establish the number of attendances per patient and referrals associated with orofacial pain and factors that may predict whether a patient is referred. A retrospective observational study was completed using the nationwide Secure Anonymised Information Linkage Databank of visits to general medical practices in Wales (UK). Data were extracted using diagnostic codes (“Read codes”). Orofacial and migraine Read codes were extracted between 1974 and 2017. Data were analyzed using descriptive statistics and univariate and multivariable logistic regression. Over the 44-y period, there were 468,827 POFP and migraine diagnostic codes, accounting for 468,137 patient attendances, or 301,832 patients. The overall attendance rate was 4.22 attendances per 1,000 patient-years (95% confidence interval [CI], 4.21–4.23). The attendance rate increased over the study period. Almost one-third of patients (*n* = 92,192, 30.54%) attended more than once over the study period, and 15.83% attended more than once within a 12-mo period. There were 20,103 referral codes that were associated with 8,183 patients, with over half these patients being referred more than once. Odds of receiving a referral were highest in females (odds ratio [OR], 1.23; 95% CI, 1.17–1.29), in those living in rural locations (OR, 1.17; 95% CI, 1.12–1.22), and in the least deprived quintile (OR, 1.39; 95% CI, 1.29–1.48). Odds also increased with increasing age (OR, 1.03; 95% CI, 1.03–1.03). The increasing attendance may be explained by the increasing incidence of POFP within the population. These patients can attend on a repeated basis, and very few are referred, but when they are, this may occur multiple times; therefore, current care pathways could be improved.

## Introduction

Orofacial pain is one of the most common causes of persistent (also previously known as chronic) pain (Breivik et al. 2006), affecting around 7% of the UK population (Aggarwal et al. 2006). Persistent orofacial pain (POFP) encompasses several conditions/disorders: temporomandibular disorders (TMDs), persistent idiopathic orofacial pain, burning mouth syndrome (BMS), posttraumatic trigeminal neuropathic pain, and trigeminal neuralgia (International Classification of Orofacial Pain 2020). The most common is TMD (Maixner et al. 2011), a collective term for musculoskeletal conditions involving pain and/or dysfunction in the muscles of mastication, temporomandibular joint, and associated structures (de Leeuw and Klasser 2018). Migraine can also present in the face and be considered a POFP diagnosis (Headache Classification Committee of the International Headache Society 2013; International Classification of Orofacial Pain 2020) and can be comorbid with other POFP diagnoses (e.g., TMD has 4 to 5 times the odds of comorbid migraine) (Réus et al. 2022). POFP also co-occurs with other persistent pain conditions (e.g., irritable bowel syndrome) and may be part of a wider spectrum of pain disorders with psychosocial comorbidity (Aggarwal et al. 2006).

POFP exerts substantial quality of life (Shueb et al. 2015) and economic impacts (Durham et al. 2016; Breckons et al. 2018). Those experiencing POFP can present to a range of health care professionals, including general dental practitioners (GDPs) and general medical practitioners (GMPs), being informally referred between the two as well as being referred to multiple secondary care services (Breckons et al. 2017). Evidence suggests that health care professionals find POFP difficult to diagnose/manage (Peters et al. 2015), leading to complex care pathways for these patients negatively affecting their pain and long-term management (Durham et al. 2010, 2021). Although attendances at GMPs for dental problems have been investigated (Anderson et al. 1999; Cope et al. 2016; Currie et al. 2022), attendances for POFP diagnoses have not.

The aim of this study was to describe 44-y trends in GMP attendances for POFP. Specific objectives were to

- Explore the number of attendances and sociodemographic factors of POFP patients
- Establish the number of referrals associated with POFP as well as factors that may predict referral

## Materials and Methods

The study details have been described in full elsewhere (Currie et al. 2022). In brief, an observational study was completed using the General Practitioner (GP) data set within the Secure Anonymised Information Linkage (SAIL) Databank (Ford et al. 2009). SAIL is a national data set comprising anonymized health and administrative data sets from Wales with over 40 y of data on Welsh GMP attendances (“GP data set”). The GP data set gave annual, cross-sectional data on patient attendances for POFP for each of the 44 y. Approval was granted by the Health Information Research Unit Information Governance Review Panel.

Data were identified and extracted by a SAIL analyst. At the time of data extraction, the data set covered 76.9% of GMP practices (further details are available in the Appendix’s narrative and Appendix Table 1). All patient attendances for POFP were included between January 1, 1974, and December 31, 2017. Identification of relevant attendances was with dental and orofacial Read codes (version 2) (Appendix Table 2). Read codes are a clinical terminology used in UK General Medical Practice based on medical terms. They include/cross-reference all other widely used medical classifications and code details of multiple demographics, investigations, therapeutics, and operative treatments of individual patients (Chisholm 1990). The reasoning for inclusion of the selected orofacial pain (OFP) Read codes is given in the Appendix. Acute dental pain Read codes were excluded, but nonspecific dental Read codes that could encompass symptoms of POFP (e.g., persistent idiopathic dentoalvaolar pain being coded as tooth symptoms) were included (Appendix Table 2). Read codes for migraine were included to encompass both migraine as a potential POFP diagnosis and as a comparator against other diagnoses.

For each Read code, the following covariates were extracted: patient ID, week of birth (actual date of birth not provided due to data protection), gender, Welsh Index of Multiple Deprivation (WIMD) quintile, Urban/Rural classification, and attendance date. WIMD is the official measure of relative deprivation (Welsh Government 2011), and the Office for National Statistics Urban/Rural classification 2001 (Office for National Statistics 2004) divides areas in urban and rural categories with further subdivisions by sparsity (further details in the Appendix). Patient age was calculated using week of birth (date of the Monday that occurred prior to, or on, their actual date of birth) and attendance date. For each POFP attendance identified, associated referral Read codes were also included. Rate of attendance was calculated as number of attendances over time and converted into attendance rates per 1,000 patient-years using the Welsh Demographic Service data set (supplemental details in the Appendix).

Data cleaning was undertaken prior to analysis with STATA v15 (StataCorp LP) within the SAIL portal. To protect patients’ confidentiality, counts fewer than 5 were not exported from the portal; therefore, Read codes were grouped into larger diagnostic groups (Appendix Table 2). Where regrouping was not possible, counts were denoted as “<5” and the total number for that variable adjusted to equal zero in subtotals. Read codes relating to nonspecific dental diagnoses/symptoms that could be suggestive of OFP (Appendix Table 2) were grouped to form a “nonspecific dental diagnosis” group. Data were analyzed using descriptive statistics, with data grouped to ensure that they were analyzed as independent observations to account for expected overlap among OFP complaints. To examine predictors of being referred, univariate and multivariable logistic regression modeling was performed. The binary response variable was whether a patient was referred or not and whether a patient received more than 1 referral or not over the study period. Explanatory variables were gender, age, WIMD, urban/rural, and potential confounders, and interactions between age, gender, WIMD, and urban/rural were assessed. Modeling was repeated with migraine excluded and with migraine only as a comparator. Regression modeling was repeated with adjustments for any potential confounders and included in the final model where a larger than 10% change was observed.

## Results

Over the period studied, there were 468,827 POFP Read codes, accounting for 468,137 patient attendances, or 301,832 patients. The overall attendance rate was 4.22 attendances per 1,000 patient-years (95% confidence interval [CI], 4.21–4.23); this reduced to 1.53 attendances per 1,000 patient-years (95% CI, 1.52–1.54) when migraine was excluded. Patients most commonly attended with migraine, followed by nonspecific dental diagnoses and TMD. The breakdown by diagnosis is given in Table 1. In total, 5,508 (1.82%) patients had a diagnosis of both migraine and TMD over the time period studied.

**Table 1.** Breakdown of Number of Attendances by Diagnosis during the 44-y Study Period, 1974 to 2017.

Diagnosis	Read Codes		Patient Attendances		Attendance Rate/1,000 Patient-Years (95% CI)
	*n*	%	*n*	%	
Migraine	298,665	63.70	298,552	63.77	2.69 (2.68–2.70)
TMD	57,800	12.33	57,419	12.27	0.52 (0.52–0.52)
Trigeminal neuralgia	19,741	4.21	19,698	4.21	0.18 (0.18–0.18)
BMS	8,291	1.77	8,275	1.77	0.07 (0.07–0.08)
Atypical facial pain	7,383	1.57	7,343	1.57	0.07 (0.06–0.07)
Postherpetic trigeminal neuralgia	1,423	0.30	1,420	0.30	0.01 (0.01–0.01)
Trigeminal nerve injury	32	0.01	32	0.01	0.0003 (0.0002–0.0004)
Nonspecific dental diagnoses	75,492	16.10	75,398	16.11	0.68 (0.68–0.69)
All	468,827	100.00	468,137	100.00	4.22 (4.21–4.42)

BMS, burning mouth syndrome; CI, confidence interval; TMD, temporomandibular disorder.

**Figure 1.**
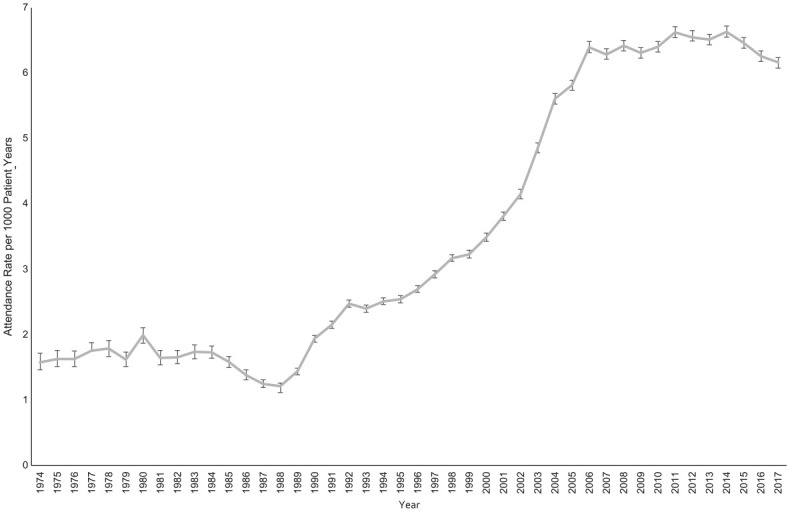
Attendance rate for all patients with persistent orofacial pain diagnoses over the 44-y study period. Number of patients = 301,832. Actual attendance rates and 95% CIs are shown.

**Figure 2.**
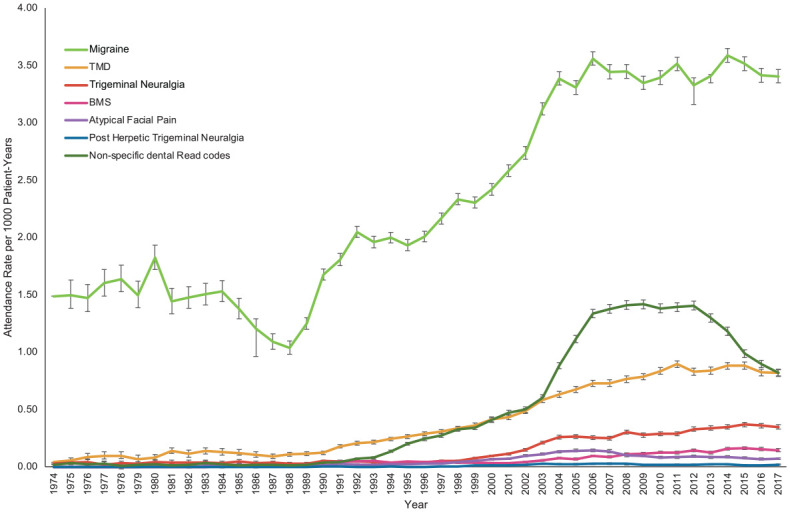
Attendance rate over the 44-y study period by persistent orofacial pain diagnosis. Number of patients = 301,832. BMS, burning mouth syndrome; TMD, temporomandibular disorder. Actual attendance rates and 95% CIs are shown.

Patient attendances for POFP increased from 1988 to 2006 and then remained relatively stable (Fig. 1). Migraine was consistently the most common diagnosis. All diagnoses demonstrated an increase in attendance rate over the study period except for nonspecific dental diagnoses, which initially increased and then declined following 2012 (Fig. 2).

Detailed patient demographics are given in Appendix Tables 3 and 4. Patients tended to be female (71.66%), with a preponderance from 20 to 29 y (20.58%) and 30 to 39 y (18.64%). The median patient age was 34.54 y. Patients were more commonly from urban areas (65.92%) and relatively equally distributed between WIMD quintiles (χ^2^ (4 *df*, *n* = 468,137) = 32.39, *P* = 0.996).

Almost one-third of patients (*n* = 92,192, 30.54%) attended more than once over the study period. In total, 47,769 patients (15.83%) attended more than once within a 12-mo period. The breakdown of number of attendances over the period studied and within 12 mo are in Table 2. The number of attendances for patients with a diagnosis of both migraine and TMD is given in Appendix Table 5.

**Table 2.** Number of Attendances for Persistent Orofacial Pain within 12 Mo and over the 44-y Study Period.

No. of Attendances	Within 12 mo		Over 44-y Study Period	
	*n*	%	*n*	%
1	254,063	84.17	209,640	69.46
2	31,551	10.45	53,232	17.64
3	8,663	2.87	19,044	6.31
4	3,317	1.10	8,591	2.85
5	1,607	0.53	4,404	1.46
6	932	0.31	2,420	0.80
7	531	0.18	1,465	0.49
8	326	0.11	871	0.29
9	218	0.07	585	0.19
10 or more	624	0.21	1,580	0.52
Total	301,832	100.00	301,832	100.00

There were 20,103 referral Read codes associated with POFP and migraine diagnostic Read codes. These were associated with 8,183 patients, with over half these patients being referred more than once (Table 3). Referral locations are given in Appendix Table 6 and included a range of health care professionals across both National Health Service (NHS) and private providers, as well as referral pathways for suspected head and neck cancer. The number of referrals by diagnosis is given in Appendix Tables 7 and 8.

**Table 3.** Number of Times Patients Were Referred Over the 44-y Study Period.

No. of Times Referred	No. of Patients	Percentage
1	3,923	47.94
2	1,599	19.54
3	915	11.18
4	601	7.34
5	372	4.55
6	259	3.17
7	185	2.26
8	87	1.06
9	153	1.87
10 or more	89	1.09

Results of the full regression analysis are given in Appendix Tables 9 to 12. Female patients were more likely to be referred for all diagnoses (odds ratio [OR], 1.23; 95% CI, 1.17–1.29; *P* < 0.0001), and increasing age had a slightly increased odds of referral for POFP diagnoses (OR, 1.03; 95% CI, 1.03–1.03; *P* < 0.0001; Appendix Tables 9 and 10), whereas increasing age had decreasing odds for migraine (Appendix Table 11). The odds of being referred varied across WIMD quintiles, with those in the least deprived quintile having the greatest odds of being referred (OR, 1.39; 95% CI, 1.29–1.48; *P* < 0.0001). Referrals were also more likely in rural locations for POFP diagnoses (OR, 1.17; 95% CI, 1.12–1.22; *P* < 0.0001) but less likely for migraine diagnoses (OR, 0.90; 95% CI, 0.86–0.95; *P* < 0.0001). Repeated referrals were associated with similar demographics (Appendix Table 12) except for living in a rural location, which decreased the odds of receiving more than 1 referral (OR, 0.86; 95% CI, 0.82–0.89; *P* < 0.0001). Increasing number of attendances decreased the odds of being referred (OR, 0.88; 95% CI, 0.87–0.89; *P* < 0.0001). There was no evidence of confounding within the multivariable regression modeling (Appendix Tables 9–12).

## Discussion

Over the study period, there was a large increase in patients seeking GMP care for POFP. Attendances for all diagnoses increased, with the most pronounced increase being migraine and TMD. Patients tended to be female, and almost one-third attended more than once. Despite the number of attendances, only a small proportion of patients were recorded as being referred for their POFP. Factors associated with being referred included being female, increasing age, and patient location.

Limitations to this study include findings relying on accurate Read code reporting by GMPs. There are no standard rules on coding in primary medical care (SAIL Databank 2020), and GMP coding behavior may therefore vary. Given the evidence suggesting that GMPs find POFP difficult to diagnose, with lack of training of GMPs in oral and dental diagnoses, there may be diagnostic errors that have translated into coding errors—for example, age breakdown for BMS (Appendix Table 4) having a large younger demographic than expected. In addition, some diagnoses may be underestimated as, for example, migraine can be misdiagnosed as sinusitis and TMD as otalgia (Kuttila et al. 2001), and some bias may have been introduced with the inclusion of Read codes such as “temporomandibular click” to ensure all POFP attendances were captured. For this reason, the data presented by diagnosis should be interpreted with caution, and rather, the data presented are best regarded as a representation of overall burden of all POFP in primary medical care. In addition, within the Read codes available, it was not possible to break diagnoses into acute and persistent diagnoses; therefore, some patients who attended only once may actually represent an acute OFP presentation (e.g., acute TMD). However, given that OFP being present for more than 3 months significantly increases the likelihood of care seeking (Macfarlane et al. 2003), this could equally represent someone with a persistent presentation seeking care from their GMP for the first time. A further limitation within the Read code classification is that diagnostic codes do not match the diagnostic criteria used for OFP; therefore, some attendances included may not have pain (e.g., nonpainful TMD subtype). Finally, the actual prevalence of orofacial pain in the Welsh general population over the study period is unknown, and it is therefore not possible to conclude whether changes observed are due to changes in prevalence or care-seeking behavior. A strength of this study is the large sample size over a long time period, meaning that issues with statistical power were not a concern.

The increase in attendance rates for POFP could be explained by increased awareness or better reporting of POFP diagnoses, increasing NHS dental costs or access issues driving patients to attend their GMP rather than GDP, and increased incidence of POFP. Given that by the end of the study period, the rate of nonspecific dental Read codes was reducing, this could perhaps reflect an increase in GMPs’ confidence in diagnosing POFP. There is evidence, however, that the prevalence and “chronification” of TMD is increasing (Häggman-Henrikson et al. 2020), and this could therefore represent an increase in number of patients seeking care as result of this.

Patients tended to be female across all diagnoses, which is in keeping with the wider literature on POFP and migraine (Macfarlane et al. 2002; Koopman et al. 2009). The age groups at presentation are largely in keeping with the expected age range for these diagnoses (Macfarlane et al. 2002; Koopman et al. 2009; Häggman-Henrikson et al. 2020). Adolescents also attended almost as frequently as young adults with TMD, supporting the suggestion that development of TMD in adolescence indicates an underlying vulnerability to musculoskeletal pain and increased likelihood of developing persistent pain from TMD into young adulthood (LeResche et al. 2007).

Patients presented from across all quintiles of WIMD with no obvious social gradient present. This is in contrast with patients presenting with acute dental pain, where there is a clear social gradient with patients from the most deprived areas being more likely to experience acute dental pain (Vargas et al. 2000; Steele et al. 2011; Currie et al. 2022). Persistent painful conditions, such as migraine (Burch et al. 2021), also tend to exhibit a social gradient, but there is mixed evidence within the POFP literature (Von Korff et al. 1988; Andersson et al. 1993; Goulet et al. 1995; Aggarwal et al. 2003; Slade et al. 2013), and it is generally agreed that there is little association between POFP and socioeconomic status. This finding therefore supports the existing literature showing lack of social gradient for POFP but is in contrast with existing literature on migraine. This could be explained by some the limitations of using WIMD in Wales where there are large rural areas where people from the most deprived areas are more geographically dispersed and more disproportionately affected by some deprivation indictors (Jones 2015).

Most patients attended their GMP only once, but it is unknown whether they had also attended elsewhere, for example, other primary care services such as their GDP or secondary care services. Alternatively, these patients could reflect the 85% of patients with persistent pain who do not need extensive treatment (Yekkalam and Wänman 2016) and as such were managed successfully by the GMP with education and self-management techniques. A proportion of patients attended on a repeated basis, with some patients having over 10 attendances. This may reflect the complex care pathways these patients go through to receive a diagnosis and manage their pain (Breckons et al. 2017; Durham et al. 2011). These repeated GMP attendances will be adding to the already established economic impact of POFP (Durham et al. 2016) and highlight the need to streamline POFP care pathways.

Despite the number of patients seeking care from their GMP, only around 3% were referred, suggesting that GMPs may feel comfortable managing these diagnoses in primary care; this contrasts with a much higher rate of referrals from GMPs for acute dental pain presentations (Currie et al. 2022) that GMPs are unable to treat. This low referral rate could contradict the previous report of GMPs feeling inadequately equipped to manage POFP patients but could be in keeping with GMPs feeling that they are obligated to treat these patients given they are able to manage patients with other long-term chronic conditions (Peters et al. 2015). Alternatively, these low referral numbers could suggest other factors such as lack of appropriate specialist services to refer patients to, which could explain the number of private referrals included. Another possibility is that these patients may have been referred but an associated Read code was not recorded, or, as previously reported (Breckons et al. 2017), they may have been informally referred to another service, such as their GDP. As discussed above, coding behavior of GMPs is important to consider, and perhaps where a patient receives an initial referral, this may not be considered important enough to code if it is documented elsewhere (e.g., in a referral letter), but the GMP may then be more likely to record subsequent referrals if patient management is becoming more complex. Regardless of this, the low number of referrals seen here, followed by the number of repeated referrals, may not result in the most optimal outcomes for patients with POFP. First, given that a failure to receive a diagnosis and appropriate management can lead to a worsening of symptoms in these patients (Breckons et al. 2017), it could be argued that patients should be referred earlier to ensure they receive this information and reassurance if the GMP feels unable to do this. Second, this failure in early referral can also lead to a breakdown in the doctor–patient relationship (Peters et al. 2015). This again suggests that care provision and pathways for these patients need to be clarified and improved. Better guidance for GMPs on decision-making in the management of these patients and encouraging and directing referrals earlier are likely to improve patient experience and outcome.

For patients who were referred, certain demographics were associated with a referral. Female patients were significantly more likely to be referred. This could be in keeping with the higher number of female patients experiencing POFP or the fact that female patients have a higher odds of being referred by GMPs for all conditions (Olthof et al. 2019). An alternative explanation could relate to gender norms, with males being less likely to seek care for pain (Keogh et al. 2000) and therefore being underrepresented. Patients presenting with POFP diagnoses tend to be young adults or middle aged, which could explain why elderly patients had a higher odds of being referred if they presented with new-onset facial pain, which could indicate a sinister underlying pathology. Adolescent patients presenting with OFP were the least likely age group to be referred despite the increased risk of “chronification” in these young patients (List et al. 2001). Patient location was also associated with receiving a referral, with patients in rural areas being more likely to be referred for all diagnoses except migraine. The reasons for this are unknown, but this could relate to dental access issues in rural areas for these patients if informal referrals between GMPs and GDPs are not possible, resulting in a referral being made at an earlier stage. This would be in keeping with the comparator of migraine, whereby living in a rural area was not a predictor of receiving a referral where dental access would not influence GMPs’ decision-making, and the fact that repeated referrals were less likely in rural areas. Finally, patients from the most deprived areas were less likely to be referred, which, given the equal spilt in presentations by WIMD, could indicate inequalities in being referred for specialist management, which may warrant further research.

In conclusion, an increasing number of patients are seeking care from their GMP for POFP. These patients can attend on a repeated basis and very few are referred, but when they are referred, this may occur multiple times. Predictors of receiving a referral for POFP include female gender, older age, and patient location.

## Author Contributions

C.C. Currie, contributed to conception, design, data acquisition, analysis and interpretation, drafted the manuscript; J. Palmer, contributed to data interpretation, drafted the manuscript; S.J. Stone, P. Brocklehurst, M.S. Pearce, J. Durham, contributed to conception, design, data acquisition and interpretation, critically revised the manuscript; V.R. Aggarwal, P.J. Dorman, contributed to data interpretation, critically revised the manuscript. All authors gave final approval and agree to be accountable for all aspects of the work.

## Supplemental Material

sj-docx-1-jdr-10.1177_00220345221128226 – Supplemental material for Persistent Orofacial Pain Attendances at General Medical Practitioners Click here for additional data file. Supplemental material, sj-docx-1-jdr-10.1177_00220345221128226 for Persistent Orofacial Pain Attendances at General Medical Practitioners by C.C. Currie, J. Palmer, S.J. Stone, P. Brocklehurst, V.R. Aggarwal, P.J. Dorman, M.S. Pearce and J. Durham in Journal of Dental Research

## References

[bibr1-00220345221128226] Aggarwal R McBeth J Zakrzewska JM Lunt M Macfarlane GJ . 2006. The epidemiology of chronic syndromes that are frequently unexplained: do they have common associated factors? Int J Epidemiol. 35(2):468–476. 16303810 10.1093/ije/dyi265

[bibr2-00220345221128226] Aggarwal VR Macfarlane TV Macfarlane GJ . 2003. Why is pain more common amongst people living in areas of low socio-economic status? A population-based cross-sectional study. Br Dent J. 194(7):383–387. 12821918 10.1038/sj.bdj.4810004

[bibr3-00220345221128226] Anderson R Richmond S Thomas DW . 1999. Patient presentation at medical practices with dental problems: an analysis of the 1996 General Practice Morbidity Database for Wales. Br Dent J. 186(6):297–300. 10230104 10.1038/sj.bdj.4800091

[bibr4-00220345221128226] Andersson HI Ejlertsson G Leden I Rosenberg C . 1993. Chronic pain in a geographically defined general population: studies of differences in age, gender, social class and pain localization. Clin J Pain. 9(3):174–182. 8219517 10.1097/00002508-199309000-00004

[bibr5-00220345221128226] Breckons M Bissett SM Exley C Araujo-Soares V Durham J . 2017. Care pathways in persistent orofacial pain: qualitative evidence from the DEEP study. JDR Clin Trans Res. 2(1):48–57. 28879244 10.1177/2380084416679648 PMC5576045

[bibr6-00220345221128226] Breckons M Shen J Bunga J Vale L Durham J . 2018. DEEP study: indirect and out-of-pocket costs of persistent orofacial pain. J Dent Res. 97(11):1200–1206. 30011387 10.1177/0022034518773310

[bibr7-00220345221128226] Breivik H Collett B Ventafridda V Cohen R Gallacher D . 2006. Survey of chronic pain in Europe: prevalence, impact on daily life, and treatment. Eur J Pain. 10(4):287–333. 16095934 10.1016/j.ejpain.2005.06.009

[bibr8-00220345221128226] Burch R Rizzoli P Loder E . 2021. The prevalence and impact of migraine and severe headache in the United States: updated age, sex, and socioeconomic-specific estimates from government health surveys. Headache. 61(1):60–68. 33349955 10.1111/head.14024

[bibr9-00220345221128226] Chisholm J. 1990. The Read clinical classification. British Medical Journal, 300(6732):1092. 2344534 10.1136/bmj.300.6732.1092 PMC1662793

[bibr10-00220345221128226] Cope AL Chestnutt IG Wood F Francis NA . 2016. Dental consultations in UK general practice and antibiotic prescribing rates: a retrospective cohort study. Br J Gen Pract. 66(646):e329–e336. 10.3399/bjgp16X684757 PMC4838445 27025554

[bibr11-00220345221128226] Currie CC Stone SJ Brocklehurst P Slade G Durham J Pearce MS . 2022. Dental attendances to general medical practitioners in Wales: a 44 year-analysis. J Dent Res. 101(4):407–413. 34582311 10.1177/00220345211044108 PMC8935529

[bibr12-00220345221128226] de Leeuw R Klasser GD , editors. 2018. Orofacial pain: guidelines for assessment, diagnosis, and management. American Academy of Orofacial Pain. 6th ed. Hanover Park (IL): Quintessence Publishing Co.

[bibr13-00220345221128226] Durham J Breckons M Vale L Shen J . 2021. DEEP study: modeling outcomes and costs of persistent orofacial pain. JDR Clin Transl Res [epub ahead of print 17 Dec 2021]. doi:10.1177/23800844211063870 PMC9773005 34915751

[bibr14-00220345221128226] Durham J Shen J Breckons M Steele JG Araujo-Soares V Exley C Vale L . 2016. Healthcare cost and impact of persistent orofacial pain: the DEEP study cohort. J Dent Res. 95(10):1147–1154. 27154734 10.1177/0022034516648088

[bibr15-00220345221128226] Durham J Steele J Moufti MA Wassell R Robinson P Exley C . 2011. Temporomandibular disorder patients’ journey through care: TMD patients’ journey through care. Community Dent Oral Epidemiol. 39(6):532–541. 21299587 10.1111/j.1600-0528.2011.00608.x

[bibr16-00220345221128226] Durham J Steele JG Wassell RW Exley C . 2010. Living with uncertainty: temporomandibular disorders. J Dent Res. 89(8):827–830. 20400717 10.1177/0022034510368648

[bibr17-00220345221128226] Ford DV Jones KH Verplancke JP Lyons RA John G Brown G Brooks CJ Thompson S Bodger O Couch T , et al. 2009. The SAIL Databank: building a national architecture for e-health research and evaluation. BMC Health Serv Res. 9:157. 19732426 10.1186/1472-6963-9-157 PMC2744675

[bibr18-00220345221128226] Goulet JP Lavigne GJ Lund JP . 1995. Jaw pain prevalence among French-speaking Canadians in Quebec and related symptoms of temporomandibular disorders. J Dent Res. 74(11):1738–1744. 8530734 10.1177/00220345950740110401

[bibr19-00220345221128226] Häggman-Henrikson B Liv P Ilgunas A Visscher CM Lobbezoo F Durham J Lövgren A . 2020. Increasing gender differences in the prevalence and chronification of orofacial pain in the population. Pain. 161(8):1768–1775. 32701837 10.1097/j.pain.0000000000001872 PMC7365674

[bibr20-00220345221128226] Headache Classification Committee of the International Headache Society. 2013. The international classification of headache disorders 3rd edition. Cephalalgia. 33(9):629–808. 23771276 10.1177/0333102413485658

[bibr21-00220345221128226] International Classification of Orofacial Pain. 2020. International classification of orofacial pain, 1st edition (ICOP). Cephalalgia. 40(2):129–221. 32103673 10.1177/0333102419893823

[bibr22-00220345221128226] Jones L . 2015. Welsh Index of Multiple Deprivation 2014: a guide to analysing deprivation in rural areas. Welsh Gov [accessed 2021 Apr 14]. https://gov.wales/sites/default/files/statistics-and-research/2019-05/welsh-index-of-multiple-deprivation-2014-a-guide-to-analysing-deprivation-in-rural-areas.pdf.

[bibr23-00220345221128226] Keogh E Hatton K Ellery D . 2000. Avoidance versus focused attention and perception of pain: differential effects for men and women. Pain. 85(1):225–230. 10692622 10.1016/s0304-3959(99)00270-5

[bibr24-00220345221128226] Koopman JSHA Dieleman JP Huygen FJ de Mos M Martin CGM Sturkenboom MCJM . 2009. Incidence of facial pain in the general population. Pain. 147(1–3):122–127. 19783099 10.1016/j.pain.2009.08.023

[bibr25-00220345221128226] Kuttila SJ Kuttila MH Niemi PM Le Bell YB Alanen PJ Suonpaa JT . 2001. Secondary otalgia in an adult population. Arch Otolaryngol Head Neck Surg. 127(4):401–405. 11296048 10.1001/archotol.127.4.401

[bibr26-00220345221128226] LeResche L Mancl LA Drangsholt MT Huang G Von Korff M . 2007. Predictors of onset of facial pain and temporomandibular disorders in early adolescence. Pain. 129(3):269–278. 17134830 10.1016/j.pain.2006.10.012 PMC1979093

[bibr27-00220345221128226] List T Wahlund K Larsson B . 2001. Psychosocial functioning and dental factors in adolescents with temporomandibular disorders: a case-control study. J Orofac Pain. 15(3):218–227. 11575192

[bibr28-00220345221128226] Macfarlane TV Blinkhorn AS Davies RM Kincey J Worthington HV . 2002. Oro-facial pain in the community: prevalence and associated impact. Community Dent Oral Epidemiol. 30(1):52–60. 11918576 10.1034/j.1600-0528.2002.300108.x

[bibr29-00220345221128226] Macfarlane TV Blinkhorn AS Davies RM Kincey J Worthington HV . 2003. Factors associated with health care seeking behaviour for orofacial pain in the general population. Community Dent Health. 20(1):20–26. 12688600

[bibr30-00220345221128226] Maixner W Diatchenko L Dubner R Fillingim RB Greenspan JD Knott C Ohrbach R Weir B Slade GD . 2011. Orofacial pain prospective evaluation and risk assessment study—the OPPERA study. J Pain. 12(11):T4–T11.e2. 10.1016/j.jpain.2011.08.002 PMC3233836 22074751

[bibr31-00220345221128226] Office for National Statistics. 2004. 2001 Rural-urban classification - Office for National Statistics. Available at: https://cy.ons.gov.uk/methodology/geography/geographicalproducts/ruralurbanclassifications/2001ruralurbanclassification (Accessed: 18 February 2020).

[bibr32-00220345221128226] Olthof M Groenhof F Berger MY . 2019. Continuity of care and referral rate: challenges for the future of health care. Fam Pract. 36(2):162–165. 29860269 10.1093/fampra/cmy048

[bibr33-00220345221128226] Peters S Goldthorpe J McElroy C King E Javidi H Tickle M Aggarwal VR . 2015. Managing chronic orofacial pain: a qualitative study of patients’, doctors’, and dentists’ experiences. Br J Health Psychol. 20(4):777–791. 25899741 10.1111/bjhp.12141

[bibr34-00220345221128226] Réus JC Polmann H Souza BDM Flores-Mir C Gonçalves DAG de Queiroz LP Okeson J De Luca Canto G . 2022. Association between primary headaches and temporomandibular disorders. J Am Dent Assoc. 153(2):120–131.e6. 10.1016/j.adaj.2021.07.021 34649707

[bibr35-00220345221128226] SAIL Databank. 2020. SAIL Databank—Primary Care GP dataset [accessed 2022 Sep 7]. https://data.ukserp.ac.uk/Asset/View/17.

[bibr36-00220345221128226] Shueb SS Nixdorf DR John MT Alonso BF Durham J . 2015. What is the impact of acute and chronic orofacial pain on quality of life? J Dent. 43(10):1203–1210. 26073033 10.1016/j.jdent.2015.06.001

[bibr37-00220345221128226] Slade GD Bair E Greenspan JD Dubner R Fillingim RB Diatchenko L Maixner W Knott C Ohrbach R . 2013. Signs and symptoms of first-onset TMD and sociodemographic predictors of its development: the OPPERA prospective cohort study. J Pain. 14(12):T20–T32.e3. 10.1016/j.jpain.2013.07.014 PMC3857109 24275221

[bibr38-00220345221128226] Steele JG Pitts N Fuller E Treasure E . 2011. Urgent conditions—a report from the Adult Dental Health Survey 2009 [accessed 2020 May 2]. https://files.digital.nhs.uk/publicationimport/pub01xxx/pub01086/adul-dent-heal-surv-summ-them-the3-2009-rep5.pdf.

[bibr39-00220345221128226] Vargas CM Macek MD Marcus SE . 2000. Sociodemographic correlates of tooth pain among adults: United States, 1989. Pain. 85(1–2):87–92. 10692606 10.1016/s0304-3959(99)00250-x

[bibr40-00220345221128226] Von Korff M Dworkin SF Le Resche L Kruger A . 1988. An epidemiologic comparison of pain complaints. Pain. 32(2):173–183. 3362555 10.1016/0304-3959(88)90066-8

[bibr41-00220345221128226] Welsh Government. 2011. Welsh Index of Multiple Deprivation 2011: Guidance on Use., GOV.WALES. Available at: https://gov.wales/sites/default/files/statistics-and-research/2019-04/wimd-2011-guidance-on-use.pdf (Accessed: 10 February 2020).

[bibr42-00220345221128226] Yekkalam N Wänman A . 2016. Factors associated with clinical decision-making in relation to treatment need for temporomandibular disorders. Acta Odontol Scand. 74(2):134–141. 26139326 10.3109/00016357.2015.1063159

