# Supplementary material for: Persistent Orofacial Pain Attendances at General Medical Practitioners
Source: J Dent Res. 2022 Oct 30;102(2):164–9. doi: 10.1177/00220345221128226 (PMC9896262; doi:10.1177/00220345221128226)
Supplement: sj-docx-1-jdr-10.1177_00220345221128226 – Supplemental material for Persistent Orofacial Pain Attendances at General Medical Practitioners [file sj-docx-1-jdr-10.1177_00220345221128226.docx]

**Persistent Orofacial Pain Attendances at General Medical Practitioners**

Currie CC, Palmer J, Stone SJ, Brocklehurst P, Aggarwal VR, Dorman PJ, Pearce MS, Durham J

**Supplemental Methodological Details**

**SAIL Cohort**

At the time of data extraction, the SAIL GP dataset covered 76.9% of GMP practices in Wales, equating to 79.4% of the Welsh population registered with a GMP practice. The population submitting data to SAIL is comparable to the general Welsh population in terms of gender, age, and Welsh Index of Multiple Depirvation (WIMD) (Appendix Table 1).

**Appendix Table 1***:* **Comparison of demographics for SAIL practices to all Welsh GMP practices.**

|  | **All Welsh Practices (%)** | **SAIL Practices (%)** |
| --- | --- | --- |
| **Gender** | | |
| Male | 49.90 | 49.86 |
| Female | 50.10 | 50.14 |
| **Age Range** | | |
| 0 to 17 years | 20.14 | 20.22 |
| 18 to 64 years | 60.28 | 60.55 |
| 65 plus years | 19.58 | 19.23 |
| **WIMD Quintile** | | |
| 1 (most deprived) | 19.09 | 20.30 |
| 2 | 18.67 | 18.53 |
| 3 | 18.64 | 18.77 |
| 4 | 17.73 | 16.19 |
| 5 (least deprived) | 18.12 | 18.61 |

**Reasoning for Inclusion of OFP Read Codes**

This study relied on both accurate diagnosis and coding of OFP diagnoses by GMPs. Given that GMPs report being unconfident in diagnosis and management of (Peters et al. 2015) this had to be considered when selecting the Read codes to extract from the SAIL Databank. For this reason all Read codes which could indicate a persistent OFP diagnosis were included. For example, “Temporomandibular click” was included on the basis that a patient with painful TMD could have a palpable click on examination, which the GMP could code.

**Welsh Index of Multiple Deprivation (WIMD)**

WIMD is the official measurement of deprivation of areas of Wales (Welsh Government, 2011) and takes into account eight different domains of deprivation: employment; income; education; health; community safety; geographical access to services; housing and physical environment. WIMD quintiles were used for analysis with quintile 1 being the 20% most deprived areas, and quintile 5 being the 20% least deprived areas of Wales.

**Office for National Statistics Urban/Rural Classification 2001**

The Office for National Statistics Urban/Rural classification 2001 (Office for National Statistics, 2016) divides geographical areas in urban (physical settlements with a population more than 10,000) and rural categories, with further subdivisions by settlement type and sparsity. Settlements in sparse areas have a particularly low number of households (compared to those defined as less sparse) and therefore may have implications on service availability.

**Calculation of Attendance Rates**

The attendance rate was calculated as the annual rate of dental consultations per 1000 patient-years to allow direct comparison with previous studies. The denominator to calculate the attendance rates was calculated using data from the Welsh Demographic Service (WDS) dataset available through SAIL. This patient level dataset contains all primary care events coded with Read codes by GMPs. This allowed calculation of the total patient-years for all attendances at all GMPs registered with SAIL by year. For partial annual data, e.g. where a patient entered the dataset part way through a calendar year, the amount of time they had contributed to that year was included by calculation of the proportion. For example, where a patient had entered the dataset in the 7th month of the year they were considered to have contributed 0.5 patient-years (6 months). Data were not available from the WDS dataset on patient location or age, therefore incidence rates could not be calculated for these variables.

**Supplemental Appendix Tables**

**Appendix Table 2:** Read codes used for data extraction and re-grouping of Read codes.

| Read Code | Read Code Description | Group |
| --- | --- | --- |
| F26 | Migraine | Migraine |
| F300 | Post herpetic trigeminal neuralgia | Post herpetic trigeminal neuralgia |
| F301z | Trigeminal neuralgia | Trigeminal neuralgia |
| F302 | Atypical facial pain | Atypical facial pain |
| J046 | Temporomandibular disorder | Temporomandibular disorders (TMDs) |
| J0462 | TMJ internal derangement |  |
| J0464 | TMJ dysfunction pain syndrome |  |
| J0463 | Temporomandibular click |  |
| S5y11 | TMJ sprain |  |
| J08zC | Burning mouth syndrome | Burning mouth syndrome (BMS) |
| J096 | Burning tongue |  |
| SJ12 | Trigeminal (5^th^) nerve injury | Trigeminal nerve injury |
| J080 | Stomatitis | Stomatitis |
| 191.. | Tooth symptoms | Non-specific dental Read codes |
| J05y | Other specified dental disorder |  |
| J052 | Dental diseases/conditions |  |

**Appendix Table 3:** Detailed patient demographics from the entire dataset (1974-2017). (WIMD = Welsh Index of Multiple Deprivation).

| **Patient Gender** | **All Diagnoses, n (%)** | **Excluding Migraine, n (%)** |
| --- | --- | --- |
| Male | 132,683 (23.34) | 59,969 (35.36) |
| Female | 335,454 (71.66) | 109,616 (64.64) |
| Indeterminate/anticipated sex change | 0 | 0 |
| Not known | 0 | 0 |
| **Patient Age Group** | **All Diagnoses, n (%)** | **Excluding Migraine, n (%)** |
| <10 years | 13,128 (2.80) | 5,897 (3.48) |
| 10-19 years | 66,334 (14.17) | 14,880 (8.77) |
| 20-29 years | 96,333 (20.58) | 29,964 (17.67) |
| 30-39 years | 87,264 (18.64) | 25,910 (15.28) |
| 40-49 years | 82,319 (17.58) | 26,408 (15.57) |
| 50-59 years | 58,312 (12.46) | 24,694 (14.56) |
| 60-69 years | 35,770 (7.64) | 20,712 (12.21) |
| 70-79 years | 20,097 (4.29) | 14,227 (8.39) |
| >80 years | 8,580 (1.83) | 6,893 (4.06) |
| **WIMD Quintile, n (%)** | **All Diagnoses, n (%)** | **Excluding Migraine, n (%)** |
| 1 (most deprived) | 96,285 (20.57) | 34,674 (20.45) |
| 2 | 89,722 (19.17) | 32,728 (19.30) |
| 3 | 96,167 (20.54) | 36,154 (21.32) |
| 4 | 87,777 (18.75) | 32,899 (19.40) |
| 5 (least deprived) | 98,186 (20.97) | 33,130 (19.54) |
| **Urban/Rural Definition, n (%)** | **All Diagnoses, n (%)** | **Excluding Migraine, n (%)** |
| Urban; sparse | 14,652 (3.13) | 6,104 (3.6) |
| Urban; less sparse | 293,928 (62.79) | 100,968 (59.54) |
| Town & fringe; sparse | 16,463 (3.52) | 7,567 (4.46) |
| Town & fringe; less sparse | 67,333 (14.38) | 24,571 (14.49) |
| Village, hamlet & isolated dwellings; sparse | 41,394 (3.13) | 17,669 (10.42) |
| Village, hamlet & isolated dwellings; less sparse | 34,367 (7.34) | 12,706 (7.49) |

**Appendix Table 4**: Detailed patient demographics by diagnosis over the entire dataset (1974-2017). **Note: counts <5 were present in subgroup analysis therefore breakdown is only shown for the most common diagnoses to protect patient confidentiality.** (TMD = Temporomandibular Disorder; BMS = Burning Mouth Syndrome; WIMD = Welsh Index of Multiple Deprivation).

|  | **Migraine** | | **TMD** | | **BMS** | | **Atypical facial pain** | |
| --- | --- | --- | --- | --- | --- | --- | --- | --- |
|  | **n** | **%** | **n** | **%** | **n** | **%** | **n** | **%** |
| **Gender** | | | | | | | |  |
| Male | 72,714 | 24.36 | 16,291 | 28.29 | 2,462 | 29.69 | 2,022 | 27.39 |
| Female | 225,838 | 75.64 | 41,509 | 71.81 | 5,829 | 70.31 | 5,361 | 72.61 |
| **WIMD** | | | | | | | |  |
| 1 | 61,611 | 20.64 | 10,764 | 18.62 | 1,571 | 18.95 | 1,288 | 17.45 |
| 2 | 56,994 | 19.09 | 10,849 | 18.77 | 1,442 | 17.39 | 1,434 | 19.42 |
| 3 | 60,013 | 20.10 | 11,552 | 19.99 | 1,736 | 20.94 | 1,554 | 21.05 |
| 4 | 54,878 | 18.38 | 10,990 | 19.01 | 1,623 | 19.58 | 1,431 | 19.38 |
| 5 | 65,056 | 21.79 | 13,645 | 23.61 | 1,919 | 23.15 | 1,676 | 22.70 |
| **Age Group** | | | | | | | |  |
| <10 | 7,231 | 2.42 | 175 | 0.30 | 533 | 6.43 | 104 | 1.41 |
| 10-19 | 51,454 | 17.23 | 8,221 | 14.22 | 421 | 5.08 | 265 | 3.59 |
| 20-29 | 66,369 | 22.23 | 11,001 | 19.03 | 601 | 7.25 | 695 | 9.41 |
| 30-39 | 61,354 | 20.55 | 9,293 | 16.08 | 775 | 9.35 | 1,113 | 15.07 |
| 40-49 | 55,911 | 18.72 | 8,972 | 15.52 | 1,099 | 13.26 | 1,451 | 19.65 |
| 50-59 | 33,618 | 11.26 | 8,147 | 14.10 | 1,351 | 16.29 | 1,457 | 19.73 |
| 60-69 | 15,058 | 5.04 | 6,547 | 11.33 | 1,604 | 19.34 | 1,164 | 15.77 |
| 70-79 | 5,870 | 1.97 | 4,007 | 6.93 | 1,259 | 15.19 | 740 | 10.02 |
| >80 | 1,687 | 0.57 | 1,437 | 2.49 | 648 | 7.82 | 394 | 5.34 |
| **Total** | 298,552 | 100.00 | 57,800 | 100.00 | 8,291 | 100.00 | 7,383 | 100.00 |

**Appendix Table 5:** Number of times patients with a diagnosis of both TMD and migraine attended over the time period studied.

| **Number of Attendances** | **Number** | **%** |
| --- | --- | --- |
| **1** | 0 | 0 |
| **2** | 1,187 | 21.55 |
| **3** | 1,136 | 20.62 |
| **4** | 821 | 14.91 |
| **5** | 570 | 10.35 |
| **6** | 437 | 7.93 |
| **7** | 264 | 4.79 |
| **8** | 234 | 4.25 |
| **9** | 163 | 2.96 |
| **10 or more** | 696 | 12.64 |
| **Total** | 5,508 | 100.00 |

**Appendix Table 6:** Breakdown of referral Read codes associated with a chronic OFP diagnosis. *NB dental referrals have been regrouped to due to counts of <5. Dental referrals were made to the following specialties: restorative dentistry; paediatric dentistry; oral surgery; endodontics; dental radiology; non-specific dental services referral.

| **Referral Code** | **Number** | **%** |
| --- | --- | --- |
| Referral needed | 8,710 | 43.33 |
| Further care referral not otherwise specified (NOS) | 3,441 | 17.12 |
| Referral to clinic | 2,900 | 14.43 |
| Referral to dental services* | 1,576 | 7.84 |
| Referral to other care | 867 | 4.31 |
| Referred to service | 590 | 2.93 |
| Referral to other clinic | 557 | 2.77 |
| Fast track suspected Head and Neck (H&N) cancer | 448 | 2.23 |
| Referral to clinic NOS | 334 | 1.66 |
| Private referral to maxillofacial surgery | 273 | 1.36 |
| Private referral to oral surgeon | 170 | 0.85 |
| Informal referral, signposted to other service | 129 | 0.64 |
| Private referral to pain management | 108 | 0.54 |
| Total | 20,103 | 100.00 |

**Appendix Table 7:** Number of referrals associated with diagnostic Read codes. NB data on referrals for post-herpetic trigeminal neuralgia and trigeminal nerve injury are excluded due to counts present <5. *indicates counts regrouped to overcome counts <5 in atypical facial pain. (TMD = Temporomandibular Disorder; BMS = Burning Mouth Syndrome).

| **Number of Referrals** | **Migraine** | **TMD** | **BMS** | **Atypical Facial Pain** | **Trigeminal Neuralgia** | **Non-specific dental** |
| --- | --- | --- | --- | --- | --- | --- |
| **1** | 2,848 | 772 | 115 | 77 | 243 | 2,006 |
| **2** | 577 | 140 | 25 | 14 | 40 | 322 |
| **3** | 219 | 43 | 9 | 8* | 14 | 109 |
| **4** | 115 | 20 | 5 | 0* | 8 | 49 |
| **5 or more** | 126 | 21 | 7 | 0* | 9 | 73 |

**Appendix Table 8:** Number of times patients with a diagnosis of both TMD and migraine were referred over the time period studied.

| **Number of Referrals** | **n** | **%** |
| --- | --- | --- |
| **0** | 5,245 | 95.23 |
| **1** | 208 | 3.78 |
| **2** | 28 | 0.51 |
| **3** | 11 | 0.20 |
| **4** | 7 | 0.13 |
| **5 or more** | 9 | 0.16 |
| **Total** | 5,508 | 100.00 |

**Appendix Table 9:** Logistic regression for referrals (all diagnoses) with adjustments in multivariable model. (WIMD = Welsh Index of Multiple Deprivation; OR = Odds Ratio).

|  | **Univariate Analysis** | | | **Adjusted for Age** | | | **Adjusted for Urban/Rural** | | | **Adjusted for Gender** | | | **Adjusted for WIMD** | | |
| --- | --- | --- | --- | --- | --- | --- | --- | --- | --- | --- | --- | --- | --- | --- | --- |
|  | **OR** | **95% CI** | **P Value** | **OR** | **95% CI** | **P Value** | **OR** | **95% CI** | **P Value** | **OR** | **95% CI** | **P Value** | **OR** | **95% CI** | **P Value** |
| **WIMD** | | | | | | | | | | | | | | | |
| 1 | 1.00 (ref) |  |  |  |  |  |  |  |  |  |  |  |  |  |  |
| 2 | 1.34 | 1.25-1.44 | <0.0001 | 1.30 | 1.21-1.39 | <0.0001 | 1.31 | 1,22-1.41 | <0.0001 | 1.34 | 1.25-1.44 | <0.0001 |  |  |  |
| 3 | 1.28 | 1.20-1.38 | <0.0001 | 1.18 | 1.10-1.27 | <0.0001 | 1.22 | 1.13-1.31 | <0.0001 | 1.28 | 1.20-1.38 | <0.0001 |  |  |  |
| 4 | 1.05 | 0.97-1.13 | 0.206 | 0.95 | 0.88-1.02 | 0.151 | 0.99 | 0.92-1.07 | 0.864 | 1.05 | 0.97-1.13 | 0.225 |  |  |  |
| 5 | 1.39 | 1.29-1.48 | <0.0001 | 1.21 | 1.13-1.30 | <0.0001 | 1.34 | 1.25-1.44 | <0.0001 | 1.38 | 1.28-1.48 | <0.0001 |  |  |  |
| **Urban/Rural** | | | | | | | | | | | | | | | |
| Urban | 1.00 (ref) |  |  |  |  |  |  |  |  |  |  |  |  |  |  |
| Rural | 1.17 | 1.12-1.22 | <0.0001 | 1.09 | 1.04-1.14 | <0.0001 |  |  |  | 1.17 | 1.12-1.22 | <0.0001 | 1.16 | 1.11-1.22 | <0.0001 |
| **Gender** | | | | | | | | | | | | | | | |
| Male | 1.00 (ref) |  |  |  |  |  |  |  |  |  |  |  |  |  |  |
| Female | 1.23 | 1.17-1.29 | <0.0001 | 1.22 | 1.16-1.28 | <0.0001 | 1.23 | 1.17-1.29 | <0.0001 |  |  |  | 1.22 | 1.16-1.29 | <0.0001 |
| **Age Group** | | | | | | | | | | | | | | | |
| <10 | 1.00 (ref) |  |  |  |  |  |  |  |  |  |  |  |  |  |  |
| 10-19 | 0.87 | 0.70-1.09 | 0.220 |  |  |  | 0.87 | 0.70-1.09 | 0.222 | 0.84 | 0.67-1.05 | 0.125 | 0.87 | 0.70-1.09 | 0.215 |
| 20-29 | 1.50 | 1.22-1.86 | <0.0001 |  |  |  | 1.51 | 1.22-1.86 | <0.0001 | 1.43 | 1.16-1.76 | <0.001 | 1.50 | 1.22-1.85 | <0.0001 |
| 30-39 | 1.99 | 1.62-2.45 | <0.0001 |  |  |  | 1.99 | 1.61-2.45 | <0.0001 | 1.89 | 1.53-2.33 | <0.0001 | 1.99 | 1.61-2.45 | <0.0001 |
| 40-49 | 2.45 | 1.99-3.01 | <0.0001 |  |  |  | 2.44 | 1.98-3.00 | <0.0001 | 2.32 | 1.88-2.86 | <0.0001 | 2.44 | 1.98-3.00 | <0.0001 |
| 50-59 | 3.43 | 2.79-4.22 | <0.0001 |  |  |  | 3.41 | 2.77-4.20 | <0.0001 | 3.26 | 2.65-4.02 | <0.0001 | 3.41 | 2.77-4.20 | <0.0001 |
| 60-69 | 4.44 | 3.60-5.47 | <0.0001 |  |  |  | 4.41 | 3.58-5.44 | <0.0001 | 4.27 | 3.47-5.27 | <0.0001 | 4.43 | 3.59-5.46 | <0.0001 |
| 70-79 | 5.34 | 4.31-6.60 | <0.0001 |  |  |  | 5.31 | 4.29-6.56 | <0.0001 | 5.14 | 4.15-6.36 | <0.0001 | 5.30 | 4.29-6.56 | <0.0001 |
| >80 | 6.89 | 5.52-8.60 | <0.0001 |  |  |  | 6.85 | 5.48-8.55 | <0.0001 | 6.59 | 5.27-8.22 | <0.0001 | 6.84 | 5.48-8.54 | <0.0001 |

**Appendix Table 10:** Logistic regression for referrals (excluding migraine) with adjustments in multivariable model. (WIMD = Welsh Index of Multiple Deprivation; OR = Odds Ratio).

|  | **Univariate Analysis** | | | **Adjusted for Age** | | | **Adjusted for Urban/Rural** | | | **Adjusted for Gender** | | | **Adjusted for WIMD** | | |
| --- | --- | --- | --- | --- | --- | --- | --- | --- | --- | --- | --- | --- | --- | --- | --- |
|  | **OR** | **95% CI** | **P Value** | **OR** | **95% CI** | **P Value** | **OR** | **95% CI** | **P Value** | **OR** | **95% CI** | **P Value** | **OR** | **95% CI** | **P Value** |
| **WIMD** | | | | | | | | | | | | | | | |
| 1 | 1.00 (ref) |  |  |  |  |  |  |  |  |  |  |  |  |  |  |
| 2 | 1.33 | 1.21-1.50 | <0.05 | 1.31 | 1.19-1.45 | <0.0001 | 1.30 | 1.18-1.44 | <0.0001 | 1.33 | 1.20-1.46 | <0.0001 |  |  |  |
| 3 | 1.18 | 1.07-1.31 | <0.001 | 1.14 | 1.03-1.26 | <0.001 | 1.12 | 1.01-1.24 | 0.032 | 1.77 | 1.07-1.30 | <0.01 |  |  |  |
| 4 | 0.96 | 0.86-1.07 | 0.443 | 0.92 | 0.83-1.02 | 0.123 | 0.90 | 0.81-1.01 | 0.073 | 0.95 | 0.86-1.06 | <0.362 |  |  |  |
| 5 | 1.36 | 1.24-1.50 | <0.0001 | 1.28 | 1.17-1.42 | <0.0001 | 1.32 | 1.20-1.46 | <0.0001 | 1.35 | 1.22-1.48 | <0.0001 |  |  |  |
| **Urban/Rural** | | | | | | | | | | | | | | | |
| Urban | 1.00 (ref) |  |  |  |  |  |  |  |  |  |  |  |  |  |  |
| Rural | 1.13 | 1.06-1.20 | <0.0001 | 1.10 | 1.04-1.17 | <0.01 |  |  |  | 1.13 | 1.06-1.20 | <0.0001 | 1.15 | 1.08-1.23 | <0.0001 |
| **Gender** | | | | | | | | | | | | | | | |
| Male | 1.00 (ref) |  |  |  |  |  |  |  |  |  |  |  |  |  |  |
| Female | 1.49 | 1.39-1.60 | <0.0001 | 1.47 | 1.38-1.58 | <0.0001 | 1.49 | 1.39-1.60 | <0.0001 |  |  |  | 1.49 | 1.39-1.59 | <0.0001 |
| **Age Group** | | | | | | | | | | | | | | | |
| <10 | 1.00 (ref) |  |  |  |  |  |  |  |  |  |  |  |  |  |  |
| 10-19 | 1.23 | 0.97-1.55 | 0.082 |  |  |  | 1.23 | 0.97-1.55 | 0.082 | 1.15 | 0.91-1.46 | 0.237 | 1.22 | 0.97-1.55 | 0.091 |
| 20-29 | 1.31 | 1.06-1.63 | 0.014 |  |  |  | 1.32 | 1.06-1.64 | <0.014 | 1.25 | 1.00-1.55 | <0.05 | 1.31 | 1.05-1.63 | <0.05 |
| 30-39 | 1.72 | 1.39-2.14 | <0.0001 |  |  |  | 1.72 | 1.34-2.14 | <0.0001 | 1.64 | 1.32-2.03 | <0.0001 | 1.71 | 1.38-2.13 | <0.0001 |
| 40-49 | 1.75 | 1.41-2.17 | <0.0001 |  |  |  | 1.74 | 1.40-2.16 | <0.0001 | 1.65 | 1.33-2.04 | <0.0001 | 1.74 | 1.40-2.15 | <0.0001 |
| 50-59 | 1.79 | 1.45-2.23 | <0.0001 |  |  |  | 1.78 | 1.44-2.21 | <0.0001 | 1.69 | 1.36-2.10 | <0.0001 | 1.78 | 1.43-2.21 | <0.0001 |
| 60-69 | 1.87 | 1.51-2.33 | <0.0001 |  |  |  | 1.86 | 1.50-2.31 | <0.0001 | 1.78 | 1.43-2.21 | <0.0001 | 1.86 | 1.49-2.31 | <0.0001 |
| 70-79 | 2.07 | 1.65-2.58 | <0.0001 |  |  |  | 2.05 | 1.65-2.56 | <0.0001 | 1.96 | 1.57-2.44 | <0.0001 | 2.04 | 1.63-2.54 | <0.0001 |
| >80 | 2.68 | 2.13-3.39 | <0.0001 |  |  |  | 2.67 | 2.12-3.37 | <0.0001 | 2.49 | 1.97-3.14 | <0.0001 | 2.65 | 2.10-3.34 | <0.0001 |

**Appendix Table 11:** Logistic regression for referrals (migraine only) with adjustments in multivariable model. (WIMD = Welsh Index of Multiple Deprivation; OR = Odds Ratio).

|  | **Univariate Analysis** | | | **Adjusted for Age** | | | **Adjusted for Urban/Rural** | | | **Adjusted for Gender** | | | **Adjusted for WIMD** | | |
| --- | --- | --- | --- | --- | --- | --- | --- | --- | --- | --- | --- | --- | --- | --- | --- |
|  | **OR** | **95% CI** | **P Value** | **OR** | **95% CI** | **P Value** | **OR** | **95% CI** | **P Value** | **OR** | **95% CI** | **P Value** | **OR** | **95% CI** | **P Value** |
| **WIMD** | | | | | | | | | | | | | | | |
| 1 | 1.00 (ref) |  |  |  |  |  |  |  |  |  |  |  |  |  |  |
| 2 | 1.35 | 1.24-1.46 | <0.0001 | 1.31 | 1.19-1.45 | <0.0001 | 1.33 | 1.22-1.44 | <0.0001 | 1.35 | 1.24-1.46 | <0.0001 |  |  |  |
| 3 | 1.37 | 1.26-1.49 | <0.0001 | 1.33 | 1.20-1.45 | <0.0001 | 1.33 | 1.23-1.49 | <0.0001 | 1.37 | 1.26-1.48 | <0.0001 |  |  |  |
| 4 | 1.13 | 1.03-1.23 | <0.01 | 1.12 | 1.02-1.22 | <0.01 | 1.09 | 1.00-1.19 | <0.05 | 1.12 | 1.03-1.22 | <0.01 |  |  |  |
| 5 | 1.47 | 1.35-1.59 | <0.0001 | 1.43 | 1.33-1.57 | <0.0001 | 1.44 | 1.33-1.56 | <0.0001 | 1.46 | 1.35-1.58 | <0.0001 |  |  |  |
| **Urban/Rural** | | | | | | | | | | | | | | | |
| Urban | 1.00 (ref) |  |  |  |  |  |  |  |  |  |  |  |  |  |  |
| Rural | 0.90 | 0.86-0.95 | <0.0001 | 0.97 | 0.92-1.02 | 0.291 |  |  |  | 0.90 | 0.85-0.95 | <0.0001 | 0.92 | 0.88-0.97 | <0.01 |
| **Gender** | | | | | | | | | | | | | | | |
| Male | 1.00 (ref) |  |  |  |  |  |  |  |  |  |  |  |  |  |  |
| Female | 1.50 | 1.41-1.59 | <0.0001 | 1.49 | 1.40-1.59 | <0.0001 | 1.50 | 1.41-1.59 | <0.0001 |  |  |  | 1.49 | 1.41-1.59 | <0.0001 |
| **Age Group** | | | | | | | | | | | | | | | |
| <10 | 1.00 (ref) |  |  |  |  |  |  |  |  |  |  |  |  |  |  |
| 10-19 | 2.86 | 2.75-2.97 | <0.0001 |  |  |  | 2.86 | 2.75-2.97 | <0.0001 | 2.85 | 2.74-2.96 | <0.0001 | 2.85 | 2.74-2.96 | <0.0001 |
| 20-29 | 1.81 | 1.75-1.89 | <0.0001 |  |  |  | 1.81 | 1.74-1.88 | <0.0001 | 1.82 | 1.75-1.89 | <0.0001 | 1.81 | 1.75-1.88 | <0.0001 |
| 30-39 | 1.88 | 1.81-1.95 | <0.0001 |  |  |  | 1.88 | 1.81-1.95 | <0.0001 | 1.88 | 1.81-1.95 | <0.0001 | 1.87 | 1.80-1.94 | <0.0001 |
| 40-49 | 1.66 | 1.60-1.72 | <0.0001 |  |  |  | 1.67 | 1.61-1.73 | <0.0001 | 1.65 | 1.61-1.73 | <0.0001 | 1.64 | 1.58-1.70 | <0.0001 |
| 50-59 | 1.05 | 1.01-1.09 | <0.001 |  |  |  | 1.06 | 1.03-1.10 | <0.001 | 1.07 | 1.03--1.10 | <0.0001 | 1.04 | 1.00-1.08 | <0.05 |
| 60-69 | 0.57 | 0.54-0.59 | <0.0001 |  |  |  | 0.57 | 0.55-0.59 | <0.0001 | 0.50 | 0.48-0.52 | <0.0001 | 0.56 | 0.53-0.58 | <0.0001 |
| 70-79 | 0.32 | 0.30-0.33 | <0.0001 |  |  |  | 0.32 | 0.31-0.34 | <0.0001 | 0.28 | 0.27-0.30 | <0.0001 | 0.31 | 0.30-0.33 | <0.0001 |
| >80 | 0.19 | 0.17-0.20 | <0.0001 |  |  |  | 0.19 | 0.18-0.20 | <0.0001 | 0.16 | 0.15-0.17 | <0.0001 | 0.18 | 0.17-0.19 | <0.0001 |

**Appendix Table 12:** Logistic regression for repeated referrals (all diagnoses) with adjustments in multivariable model. (WIMD = Welsh Index of Multiple Deprivation; OR = Odds Ratio).

|  | **Univariate Analysis** | | | **Adjusted for Age** | | | **Adjusted for Urban/Rural** | | | **Adjusted for Gender** | | | **Adjusted for WIMD** | | |
| --- | --- | --- | --- | --- | --- | --- | --- | --- | --- | --- | --- | --- | --- | --- | --- |
|  | **OR** | **95% CI** | **P Value** | **OR** | **95% CI** | **P Value** | **OR** | **95% CI** | **P Value** | **OR** | **95% CI** | **P Value** | **OR** | **95% CI** | **P Value** |
| **WIMD** | | | | | | | | | | | | | | | |
| 1 | 1.00 (ref) |  |  |  |  |  |  |  |  |  |  |  |  |  |  |
| 2 | 1.34 | 1.25-1.44 | <0.0001 | 1.30 | 1.21-1.40 | <0.0001 | 1.31 | 1.22-1.41 | <0.0001 | 1.34 | 1.25-1.44 | <0.0001 |  |  |  |
| 3 | 1.28 | 1.20-1.38 | <0.0001 | 1.18 | 1.10-1.27 | <0.0001 | 1.22 | 1.13-1.31 | <0.0001 | 1.28 | 1.20-1.38 | <0.0001 |  |  |  |
| 4 | 1.05 | 0.97-1.13 | 0.206 | 0.95 | 0.88-1.03 | 0.189 | 0.99 | 0.92-1.07 | 0.864 | 1.05 | 0.97-1.13 | 0.225 |  |  |  |
| 5 | 1.38 | 1.29-1.48 | <0.0001 | 1.32 | 1.23-1.41 | <0.0001 | 1.34 | 1.25-1.44 | <0.0001 | 1.38 | 1.28-1.48 | <0.0001 |  |  |  |
| **Urban/Rural** | | | | | | | | | | | | | | | |
| Urban | 1.00 (ref) |  |  |  |  |  |  |  |  |  |  |  |  |  |  |
| Rural | 0.86 | 0.82-0.89 | <0.0001 | 0.92 | 0.88-0.96 | <0.0001 |  |  |  | 0.85 | 0.82-0.89 | <0.0001 | 0.86 | 0.82-0.90 | <0.0001 |
| **Gender** | | | | | | | | | | | | | | | |
| Male | 1.00 (ref) |  |  |  |  |  |  |  |  |  |  |  |  |  |  |
| Female | 1.22 | 1.17-1.29 | <0.0001 | 1.22 | 1.15-1.28 | <0.0001 | 1.23 | 1.17-1.29 | <0.0001 |  |  |  | 1.22 | 1.16-1.29 | <0.0001 |
| **Age Group** | | | | | | | | | | | | | | | |
| <10 | 1.00 (ref) |  |  |  |  |  |  |  |  |  |  |  |  |  |  |
| 10-19 | 0.87 | 0.70-1.09 | 0.220 |  |  |  | 0.87 | 0.70-1.09 | 0.222 | 0.84 | 0.67-1.05 | 0.125 | 0.87 | 0.70-1.09 | 0.215 |
| 20-29 | 1.50 | 1.22-1.86 | <0.0001 |  |  |  | 1.51 | 1.22-1.86 | <0.0001 | 1.43 | 1.16-1.76 | <0.005 | 1.50 | 1.22-1.85 | <0.0001 |
| 30-39 | 1.99 | 1.62-2.45 | <0.0001 |  |  |  | 1.99 | 1.61-2.45 | <0.0001 | 1.89 | 1.53-2.33 | <0.0001 | 1.99 | 1.61-2.45 | <0.0001 |
| 40-49 | 2.45 | 1.99-3.01 | <0.0001 |  |  |  | 2.44 | 1.98-2.00 | <0.0001 | 2.32 | 1.88-2.86 | <0.0001 | 2.44 | 1.98-3.00 | <0.0001 |
| 50-59 | 3.43 | 2.79-4.22 | <0.0001 |  |  |  | 3.41 | 2.77-4.20 | <0.0001 | 3.26 | 2.65-4.02 | <0.0001 | 3.41 | 2.77-4.20 | <0.0001 |
| 60-69 | 4.44 | 3.61-5.47 | <0.0001 |  |  |  | 4.41 | 3.58-5.44 | <0.0001 | 4.27 | 4.15-6.36 | <0.0001 | 4.43 | 3.59-5.46 | <0.0001 |
| 70-79 | 5.33 | 4.31-6.60 | <0.0001 |  |  |  | 5.31 | 4.28-6.56 | <0.0001 | 5.14 | 4.15-6.36 | <0.0001 | 5.30 | 4.29-6.56 | <0.0001 |
| >80 | 6.89 | 5.51-8.59 | <0.0001 |  |  |  | 6.85 | 5.49-8.55 | <0.0001 | 6.59 | 5.27-8.22 | <0.0001 | 6.84 | 5.48-8.54 | <0.0001 |
